# Supplementary figures and images for: Predicting treatment response to cognitive behavior therapy in social anxiety disorder on the basis of demographics, psychiatric history, and scales: A machine learning approach
Source: PLoS One. 2025 Mar 18;20(3):e0313351. doi: 10.1371/journal.pone.0313351 (PMC11918322; doi:10.1371/journal.pone.0313351)

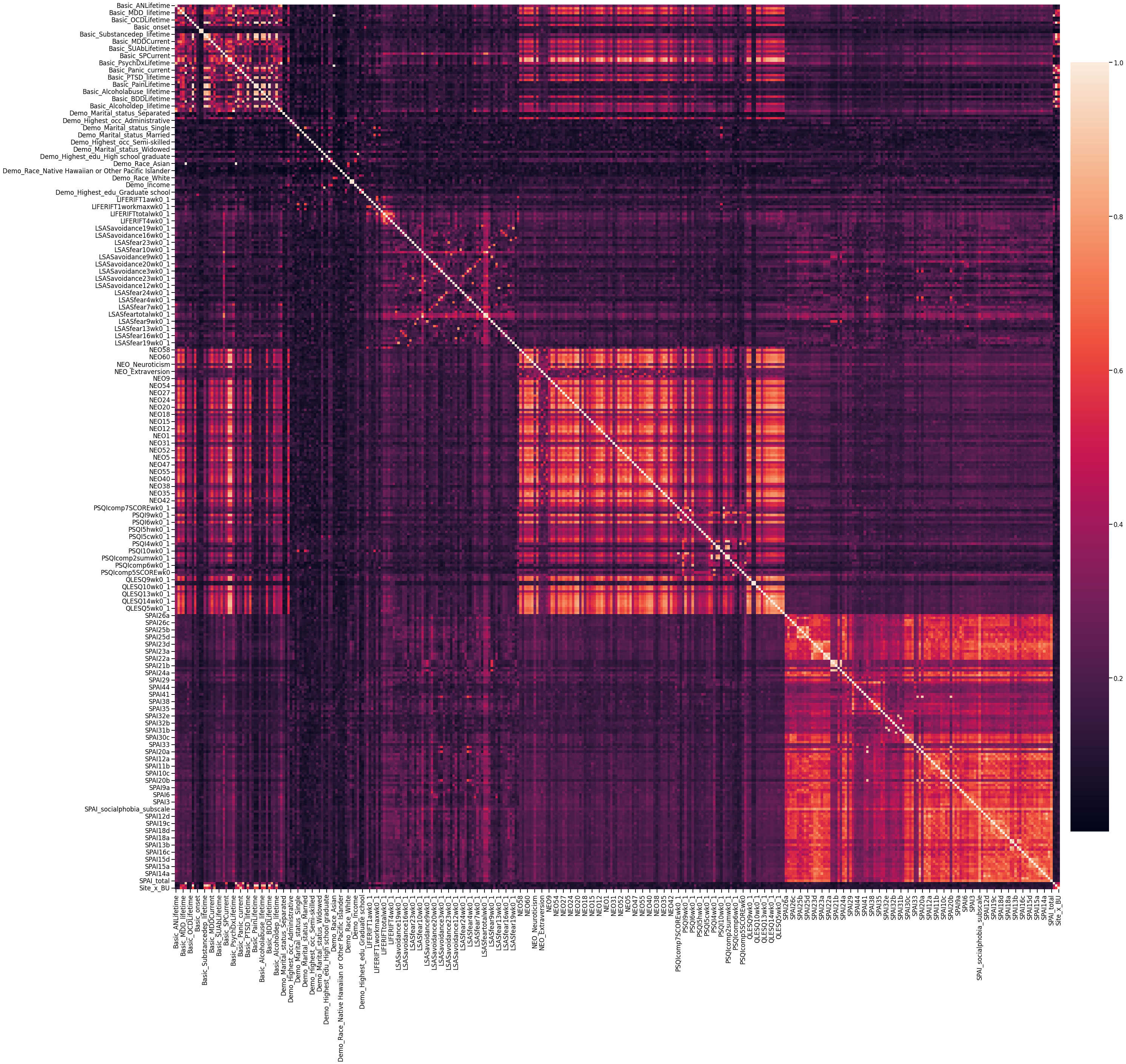

Supplement: S1 Data — S1 Table. List of all features grouped in to their respective subsets. Table containing all the features used in the analysis and their groups into respective subsets are given in the excel file. S2 Table. Weighted mean rank of features from all scales, subscales and questions derived from SHAP values. S3 Table. Explained variance of features subsets. S4 Table. Weighted mean rank of features from LSAS scales, subscales and questions derived from SHAP values. S1 Fig. Distance correlations of all features grouped according to categories. S1 Fig in S1 Data shows the relationship across all features by estimating the distance correlations and grouping them by the categories of questions. This represents the relation between various scales. (ZIP) [file pone.0313351.s001.zip › SupplementaryMaterial/SupplementaryFigure1_correlation-distance.png]
