# Supplementary material for: Predicting treatment response to cognitive behavior therapy in social anxiety disorder on the basis of demographics, psychiatric history, and scales: A machine learning approach
Source: PLoS One. 2025 Mar 18;20(3):e0313351. doi: 10.1371/journal.pone.0313351 (PMC11918322; doi:10.1371/journal.pone.0313351)
Supplement: Attachment — Submitted filename: Journal_PlosOne-Oct21QB.docx [file pone.0313351.s004.docx]

**Response to the Editor**

1. We have included the response to the reviewer file
2. We have made the code available. Data contains potentially sensitive patient information, including detailed demographic information which may identify individuals. Data will be made available to individual authors upon request from Jill Crittenden (jrc@mit.edu).

https://mcgovern.mit.edu/tile/jill-crittenden-2/

**Reviewers' comments:**

**Reviewer's Responses to Questions**

**Comments to the Author**

Reviewer #1: I believe the authors have addressed and responded well to all the critique provided by the reviewers, to the best of their capacity and given the limitations of the study.

Reviewer #2: The authors have largely been responsive and most prior concerns have been addressed. The one lingering issue is that it's not clear how clinicians, or researchers, are meant to use these findings moving forward. **The authors highlight that adding subscores and individual items to a model containing the total LSAS scores improves prediction. It would be helpful if the authors could, in plain language, explain how someone who wanted to make use of these findings should go about doing so.**

We have added the following paragraph to the discussion section to address the reviewers’ comment:

“Clinicians and researchers typically use overall summary scores from self-report questionnaires to evaluate diagnoses or response to treatment. Indeed, such summary scores typically have the most reliability and validity relative to subscores or individual items. In the present study, however, prediction was most accurate when data from individual items, subscores, and the total score were all included. A similar benefit of including all data from a single clinical questionnaire for ML prediction was observed for a study predicting the development of bipolar disorder in children over a 10-year period [56]. At a practical level, clinicians and researchers would not be expected to implement ML algorithms to calculate likelihood of treatment efficacy (or development of a diagnosis), but rather risk calculators would need to be made available that would automatically compute such probabilistic outcomes. Such risk calculators are already in use in other areas of medicine, but not yet for psychiatric diagnoses or treatments.”
